# Family Physicians’ Perspectives on Personalized Cancer Prevention: Barriers, Training Needs, Quality Improvements and Opportunities for Collaborative Networks

**DOI:** 10.3390/jcm14197073

**Published:** 2025-10-07

**Authors:** Delia Nicoara, Cosmin Cristescu, Ioan Constantin Pop, Radu Alexandru Ilies, Niculina Nicoara, Alexander Olivier von Stauffenberg, Stefan Matei, Maximilian Vlad Muntean, Patriciu Achimas-Cadariu

**Affiliations:** 1 Faculty of Medicine, “Iuliu Hațieganu” University of Medicine and Pharmacy, 400012 Cluj-Napoca, Romania; drdelianicoara@gmail.com (D.N.); ilies.radu.alexandru@elearn.umfcluj.ro (R.A.I.); 2 “Prof. Dr. I. Chiricuță” Institute of Oncology, 400015 Cluj-Napoca, Romania; niculina.nicoara@iocn.ro (N.N.); alexander.olivier@iocn.ro (A.O.v.S.); stefan.matei@iocn.ro (S.M.); 3 National Center of Competence in the field of Cancer, “Prof. Dr. I. Chiricuță” Institute of Oncology, 400015 Cluj-Napoca, Romania; pachimas@umfcluj.ro; 4 Department of Plastic Surgery, “Prof. Dr. I. Chiricuță” Institute of Oncology, 400015 Cluj-Napoca, Romania; maximilian.muntean@iocn.ro; 5 Department of Plastic and Reconstructive Surgery, “Iuliu Hațieganu” University of Medicine and Pharmacy, 400012 Cluj-Napoca, Romania; 6 Department of Surgical Oncology, “Prof. Dr. I. Chiricuță” Institute of Oncology, 400015 Cluj-Napoca, Romania; 7 Department of Surgical Oncology and Gynecologic Oncology, “Iuliu Hațieganu” University of Medicine and Pharmacy, 400012 Cluj-Napoca, Romania

**Keywords:** cancer prevention, family physicians, primary care, personalized prevention, early detection, health literacy, medical education

## Abstract

**Background/Objectives**: Family physicians are key stakeholders in the implementation of cancer prevention strategies, including risk factor assessment, lifestyle counseling, and early detection. Despite this, integration of personalized prevention into routine practice remains limited. This study aimed to explore family physicians’ perspectives on barriers, training needs, and collaboration opportunities in cancer prevention. **Methods**: A mixed-methods study was conducted using an exploratory sequential design. The qualitative phase involved semi-structured interviews with 12 family physicians from the North-West Region of Romania. Thematic analysis was employed to identify main challenges and opportunities. Findings informed the development of a structured online survey completed by 50 family physicians. Descriptive and comparative statistical analyses were applied to assess trends and subgroup differences. **Results**: Interviews and survey data revealed multiple barriers to cancer prevention in primary care: insufficient consultation time, limited access to diagnostic tools, administrative workload, and low patient health literacy. Physicians reported moderate familiarity with personalized prevention but expressed strong interest in further training, particularly through flexible and interactive learning formats. Collaboration with cancer centers was considered suboptimal; participants emphasized the need for streamlined referral pathways and improved communication. **Conclusions**: The study highlights systemic and educational gaps affecting cancer prevention efforts in family medicine. Tailored training programs, digital integration with cancer centers, and targeted policy adjustments are needed to enhance prevention capacity within primary care.

## 1. Introduction

Cancer remains one of the leading public health challenges worldwide, imposing a significant burden on healthcare systems, economies, and individuals. According to the Global Cancer Observatory, an estimated 19.3 million new cancer cases and nearly 10 million cancer-related deaths were recorded globally in 2020. The statistics indicate a concerning trend, with cancer incidence expected to increase, particularly in low- and middle-income countries due to rapid urbanization, changing lifestyles, and inadequate healthcare resources [1]. In Europe, disparities in cancer outcomes are evident, with Eastern and Central European countries facing a higher burden of disease compared to their Western counterparts, underscoring the urgent need for effective public health strategies [2,3]. Several European initiatives have recently aimed to address these challenges, including JANE (Joint Action on Networks of Expertise), JAPreventNCD (Joint Action on Prevention of Non-Communicable Diseases), PROPHET (Personalized Prevention Roadmap for the Future of Health in Europe), and EUCanScreen (European Joint Action on Cancer Screening), all of which promote collaborative frameworks and expertise networks to strengthen cancer prevention across countries [4,5,6].

A substantial proportion of cancers—including those of the breast, colorectal, lung, and cervical types—are preventable, primarily through lifestyle modifications, vaccination, and early detection strategies. Evidence suggests that approximately 30–50% of cancer cases could be avoided by adhering to risk-reduction practices [7]. Family physicians (FPs) play a critical role in cancer prevention, acting as the frontline in healthcare where they can initiate preventive measures, educate patients about risk factors, and encourage appropriate screening tests. Their extensive patient relationships and ability to provide personalized care position them uniquely to influence patients’ health behaviors positively [8,9].

Emerging solutions in cancer prevention increasingly include the integration of omics and personalized medicine approaches, which promise to refine risk assessment and tailor prevention strategies to individual profiles. These advanced methodologies leverage insights from genetics, proteomics, and metabolomics to identify patients at higher risk of cancer, allowing for targeted interventions that further improve preventive outcomes [10]. Furthermore, utilizing omics data can enhance the accuracy of family physicians in recommending appropriate screening tests and risk-reducing strategies while also improving the ability to relay complex health information to patients.

However, the effectiveness of family physicians in implementing these strategies depends significantly on adequate training and education. Continuous professional development in cancer prevention and the latest research findings is essential for empowering FPs to effectively communicate preventive measures and risk factors to their patients [11,12]. Despite their acknowledged importance, studies have indicated that many family physicians express a need for further training in cancer prevention strategies and the use of emerging technologies in their practice [13].

While the literature provides extensive data on the burden of cancer and on possible solutions, few studies explore the full continuum from identifying problems to developing solutions from the perspective of family physicians. Addressing this knowledge gap is essential for linking barriers with practical strategies in primary care. Therefore, this study aimed to investigate family physicians’ perspectives on barriers to cancer prevention, their training needs, and opportunities for collaboration with cancer centers, using a mixed-methods approach.

## 2. Materials and Methods

### 2.1. Study Design

This research employed a mixed-methods approach with an exploratory sequential design. In the first phase, qualitative data were collected and analyzed to gain in-depth insights, and these findings were then used to inform the development of a quantitative survey in the second phase. We chose this mixed-methods design because the qualitative phase allowed for an in-depth exploration of family doctors’ perspectives and experiences, while the subsequent survey enabled a broader assessment of patterns and subgroup differences, together providing a more comprehensive understanding of the research problem.

### 2.2. Qualitative Phase: Semi-Structured Interviews

#### 2.2.1. Participants and Recruitment

The qualitative phase consisted of semi-structured interviews with family physicians practicing in Cluj County (Northwest Romania) who were contracted with the Cluj County Health Insurance House, conducted between October and December 2023. Eligible doctors were first contacted by email, which included an overview of the study, the interview topics, and the estimated interview duration. Those who agreed to participate were scheduled for an interview, and a snowball sampling strategy was then used to identify additional participants (i.e., interviewed doctors referred colleagues who might provide valuable insights). A total of 12 family doctors participated in this phase, which was considered sufficient for thematic saturation (no new themes were emerging by the 12th interview). All interviews were conducted in Romanian at a time and place convenient for participants—either in person at the clinic, via videoconference, or by phone—and each interview lasted approximately 30 min. Prior to starting, participants provided informed consent, and permission was obtained to audio-record the conversation for accuracy. A semi-structured interview guide, adapted from published guidelines by DeJonckheere & Vaughn (2019) [14] was used to ensure all key topics were covered. The full list of interview questions is provided in Appendix A. This guide outlined broad questions and prompts about personalized prevention in primary care and collaboration with cancer centers, while allowing flexibility to probe further based on each participant’s responses. Semi-structured interviews were chosen because they facilitate a focused yet open-ended dialog, enabling the researcher to explore participants’ thoughts, feelings, and experiences in depth. All interviews were transcribed verbatim for analysis.

#### 2.2.2. Qualitative Data Analysis

The interview transcripts were analyzed using thematic analysis to identify recurring patterns and themes in the data. We followed a primarily deductive analytic approach, using the predefined concepts from the interview guide as initial coding categories. This means that our coding started with expected themes based on the interview topics, but we remained open to novel insights; any content that did not fit the initial categories was coded as a new theme to ensure important unexpected findings were not overlooked. To enhance reliability, transcripts were independently coded by two researchers, and discrepancies were resolved through discussion until consensus was reached. The research team familiarized themselves with the transcripts through repeated reading and then coded the data manually, grouping similar ideas under the relevant themes. Through this iterative and collaborative process of coding, reviewing, and refining, key themes were finalized to capture the range of family doctors’ perspectives. Representative quotations for each theme were identified to illustrate the findings (to be presented in the Results section).

### 2.3. Quantitative Phase: Online Survey

#### 2.3.1. Instrument Development and Data Collection

In the second phase, a structured questionnaire was developed based on the themes and insights obtained from the interviews. This approach ensured that the survey addressed all relevant issues raised during the qualitative phase, thus aligning the quantitative measures with participants’ real-world experiences. Responses were collected between January and March 2025. The questionnaire (administered via Google Forms) included sections on respondents’ demographics and practice characteristics (e.g., practice location—urban or rural, years of professional experience), as well as items examining their views and practices regarding personalized prevention and collaboration with cancer centers. It also covered aspects of ongoing professional education, such as types of training or informational resources accessed, and perceived training needs in cancer prevention. Most questions were close-ended (multiple choice or Likert-scale) to facilitate quantitative analysis, though a few open-ended questions were included to allow additional comments. The survey link was distributed by email to the same target population of family doctors in Cluj County. Participation was voluntary, and clicking the consent confirmation in the form (agreeing to participate and confirming one’s role as a family doctor) was required before proceeding to the questionnaire. Responses were collected anonymously through Google Forms over the data collection period. Once the survey was closed, data were downloaded into Microsoft Excel for initial cleaning and then imported into IBM SPSS for analysis.

#### 2.3.2. Quantitative Data Analysis

We used descriptive statistics to summarize the survey data. Categorical variables were described by frequencies and percentages, while continuous variables (if any) were summarized by measures of central tendency and dispersion (mean, median, and standard deviation). These descriptive analyses provided a profile of the sample and an overview of family doctors’ practices and needs regarding cancer prevention training.

Associations between categorical variables (e.g., practice location and type of training resources accessed) were tested using chi-square analyses. For continuous outcomes, independent-samples *t*-tests were applied for two-group comparisons, while one-way ANOVA was used for comparisons involving more than two groups. Statistical significance was defined as *p* < 0.05, and 95% confidence intervals (95% CI) were calculated where appropriate to indicate the precision of estimates. Subgroup analyses were conducted to explore the differences in training needs by professional experience. All quantitative analyses were performed using IBM SPSS Statistics (Version 30), and results are reported with corresponding *p*-values and confidence intervals to support interpretation.

### 2.4. Ethical Considerations

Ethical review and approval were waived for this study because it involved no clinical interventions, posed minimal risk, and collected only anonymized opinions from adult healthcare professionals (family physicians), with no patient data or identifiable personal information recorded. A formal waiver decision was also issued by the Ethics Committee of “Prof. Dr. I. Chiricuță” Institute of Oncology (Assessment report no. 348/12.08.2025, Application no. 7501/11.08.2025). All participants provided informed consent to partake in the research. For the interview phase, oral consent was obtained from each physician prior to their interview. In the survey phase, participants were presented with an electronic informed consent statement at the start of the Google Forms questionnaire and indicated their agreement before proceeding. Participation in both phases was entirely voluntary, and participants could decline to answer any question or withdraw at any time. We safeguarded participants’ confidentiality throughout the study: no identifying personal information was collected in the survey, and interview data were anonymized during transcription (e.g., using codes or pseudonyms for participants and clinics). All data were stored securely and analyzed in aggregate form. The study adhered to ethical principles of research with human subjects, including confidentiality and data protection. In handling survey responses and interview transcripts, we complied with the European Union’s General Data Protection Regulation (GDPR) (EU 2016/679) to ensure that personal data were processed lawfully and confidentially.

## 3. Results

### 3.1. Qualitative Findings (Thematic Analysis of Interviews)

A total of 12 family physicians were interviewed. The analysis of interview transcripts yielded several key themes regarding the role of family doctors in cancer prevention, perceived barriers, training needs, and collaboration with specialized cancer centers. The findings are presented below by theme, with illustrative quotations from participants (translated to English) to exemplify each theme.

#### 3.1.1. Emphasis on Prevention vs. Time Constraints in Practice

Family doctors unanimously acknowledged preventive care as a fundamental part of their role, yet many noted that in daily practice prevention often takes a secondary place to acute care demands. Several physicians described an ideal role where the family doctor “evaluates risk factors and counsels patients” (Interview participant) and serves as the “first line” for patient education. However, they reported that heavy workloads and limited consultation time make this difficult: “In theory, the family doctor should carry out more prevention, but unfortunately in practice a lot of time is allocated to acute pathology” (Interview participant). One doctor explained that due to many patients waiting, consultations focus on the immediate complaint, leaving little time for additional preventive counseling: “The main reason we do not [provide more prevention counseling] is lack of time… we have patients waiting and we try to solve their issue as quickly as possible” (Interview participant).

#### 3.1.2. Patient Engagement and Communication Challenges

Participants reported multiple patient-related barriers that impede effective cancer prevention. Low health literacy and misinformation were frequently mentioned. Many patients arrive with preconceived (often inaccurate) notions from the internet or acquaintances, which family doctors must address. “There is a lot of disinformation online, making it very difficult to change patients’ beliefs,” noted one physician (Interview participant). Several doctors observed that patients tend to delay seeking care and even resist preventive interventions. For example, a physician described that “in the case of patients with suspected cancer, most refuse to believe they could have cancer and refuse investigations out of fear of the diagnosis” (Interview participant). Such fear and denial, combined with cultural taboos (e.g., around discussing certain health topics), can hinder open communication. Additionally, participants felt that lack of patient motivation and financial constraints lead some individuals to decline screening tests, especially if those require out-of-pocket payment. As one doctor remarked, many patients are “reluctant to undergo tests that are not covered by insurance” (Interview participant).

#### 3.1.3. Systemic Barriers to Cancer Prevention in Primary Care

Beyond patient factors, family doctors identified several system-level obstacles limiting their preventive activities. A common concern was the healthcare system’s reimbursement and organizational structure, which does not strongly support prevention. Participants explained that preventive services are often not distinctly financed or are burdensome to bill. One physician noted that the “current method of reimbursing prevention services is not encouraging—some colleagues had their prevention service claims rejected, which discourages offering these services” (Interview participant). Doctors also pointed to bureaucratic workloads and administrative reporting that consume considerable time (often 25% or more of their workday), thereby reducing time available for prevention. Limited access to specialized investigations was another barrier, particularly in rural areas. “Unfortunately, access to specialist investigations is limited in certain geographic zones, especially rural areas,” one participant explained, noting that even when family doctors refer patients, long wait times for tests (e.g., screening mammograms) can discourage follow-through. Some doctors felt that the lack of a clear referral pathway for preventive care (e.g., no diagnostic coding for prevention referrals) makes it harder to seamlessly connect patients with cancer screening services.

#### 3.1.4. Need for Guidelines and Training in Cancer Prevention

Nearly all interviewed physicians expressed a need for more education, guidelines, and training specifically focused on cancer prevention. Many felt that staying current with evolving evidence (such as new screening methods or genetic risk markers) is challenging given their busy schedules. They welcomed support in the form of up-to-date clinical guidelines or protocols tailored for primary care. One doctor suggested that “a practical guide defining which preventive activities the family doctor should do and when to refer the patient would help a lot”, adding that often they are unsure how far to go before specialist referral (Interview participant). Another physician echoed the value of additional training opportunities, stating that such training would be useful because family doctors “do not have the necessary time to document themselves on oncology prevention” (Interview participant). Several participants recommended more organized continuing medical education—for instance, “training sessions on new information in the field (with geneticists, epidemiologists, etc.)” (Interview participant)—and even suggested that formal residency programs in family medicine allocate more time to prevention and oncology.

#### 3.1.5. Collaboration and Communication with Cancer Centers

The interviews underscored a strong perceived need for better collaboration between primary care and oncology specialists (including cancer centers). Family physicians reported that communication with specialists is often fragmented. It was common for participants to mention that when they refer patients to oncologists or other specialists, they rarely receive feedback or follow-up information. “In many situations patients we send to specialists do not bring back the results—we have no feedback from the specialist,” one doctor explained (Interview participant). Physicians felt that a more integrated approach would improve patient care continuity, suggesting solutions like direct lines of communication or shared electronic platforms to exchange patient information. Another participant emphasized ensuring timely access for patients referred to cancer centers and greater openness from specialists toward collaborating with family doctors, particularly to support personalized prevention planning. Family doctors believed that strengthening the link with cancer centers—for example, via regular meetings or a formal network—would help them stay informed about the latest recommendations and facilitate quicker referrals for high-risk patients.

### 3.2. Quantitative Findings (Survey Results)

Fifty family doctors completed the survey on personalized cancer prevention and collaboration with cancer centers. Respondents were predominantly female (43 out of 50, 86%) and mostly practiced in urban areas (36, 72%), with the remainder in rural settings. The sample was experienced: 78% had over 10 years of practice (34% between 10 and 20 years; 44% over 20 years), while 16% had less than 5 years. The results of the survey are organized below by key topics, including physicians’ current preventive practices and perspectives, perceived barriers to providing preventive services, identified training needs, and views on collaboration with cancer centers.

#### 3.2.1. Current Practices and Level of Knowledge in Cancer Prevention

Most family doctors reported being moderately familiar with the concept of personalized prevention, though full expertise was uncommon. Over half (56%) rated their understanding of personalized cancer prevention as “moderate,” and only 12% felt they knew it “very well.” In contrast, familiarity with emerging “omic” sciences (such as genetics or proteomics relevant to prevention) was generally low—none of the respondents indicated being very familiar with omic concepts, and the majority (54%) admitted having only a little knowledge in this area. Awareness of established guidelines like the European Code Against Cancer was mixed: while 18% were largely familiar with the recommendations and 4% felt completely up-to-date, about half (48%) were only partially familiar and 30% not at all familiar with the Code’s recommendations.

In terms of current prevention activities, most surveyed physicians consistently incorporate lifestyle counseling into routine care. Forty-three doctors (86%) reported that they very frequently advise patients on lifestyle modifications (such as diet, exercise, smoking cessation), and the remaining 14% do so at least occasionally. However, the utilization of advanced tools for risk stratification is limited. Polygenic risk scores (which assess genetic risk based on multiple genes) were rarely used in practice—70% of respondents have never used such scores, and none reported using them regularly with most patients. When asked about recommending newer types of preventive tests to patients, responses indicated a selective approach. A majority (62%) had at some point recommended proteomic biomarker tests (for example, cancer antigen tests like CA-125 for early detection), making this the most used category of “omics” testing in primary care. In contrast, only 24% had recommended genetic tests (such as BRCA1/2 screening for hereditary cancer risk), and just 22% had ever suggested microbiome analyses as part of prevention. Virtually, no one reported ordering epigenetic tests (only 1 physician, 2% of the sample, had done so).

Most respondents had some firsthand experience with organized prevention or screening programs. In fact, 78% reported having participated in either a primary prevention project or a cancer screening program in the past. Specifically, 38% had been involved in primary prevention initiatives (such as community prevention campaigns), 48% in screening programs (e.g., organized screening for cancers), and 8% had experience with both types of projects. Only about one-fifth (22%) had never taken part in such preventive projects.

#### 3.2.2. Barriers to Preventive Services

**Figure 1:**
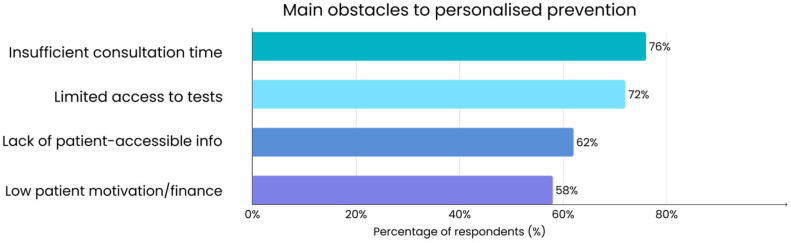
Main obstacles to implementing personalized cancer prevention reported by surveyed family doctors (N = 50). Multiple responses were allowed for this survey question, so percentages do not sum to 100%.

The survey asked physicians to identify the primary obstacles they face in implementing personalized prevention for cancer in their practice. As shown in Figure 1, the most common barrier identified by respondents was insufficient consultation time, followed by limited access to personalized diagnostic tests. Physicians also noted the lack of clear and accessible patient information, which makes it difficult to support preventive measures in practice. Low patient motivation and financial constraints were mentioned as additional obstacles.

#### 3.2.3. Training Needs and Preferences

**Figure 2:**
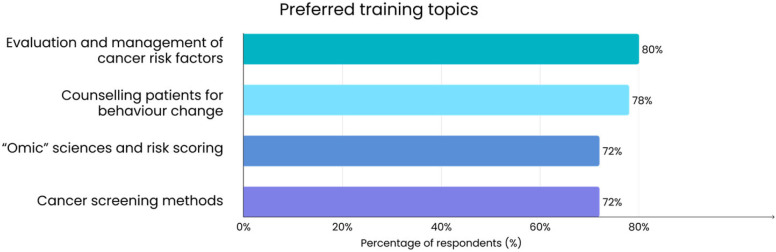
Preferred training topics among surveyed family doctors (*n* = 50). Multiple responses were allowed for this survey question, so percentages do not sum to 100%.

The survey results indicated a very high interest in further training on cancer prevention among family doctors. Nearly all respondents (94%) answered “Yes” when asked if they would like to participate in training programs on personalized prevention and screening. Doctors also reported which training topics they would like to address in more depth. As shown in Figure 2, the most frequently selected areas were the evaluation and management of cancer risk factors, methods for counseling patients on behavior change, and emerging domains such as “omic” sciences, risk scoring, and cancer screening methods.

**Figure 3:**
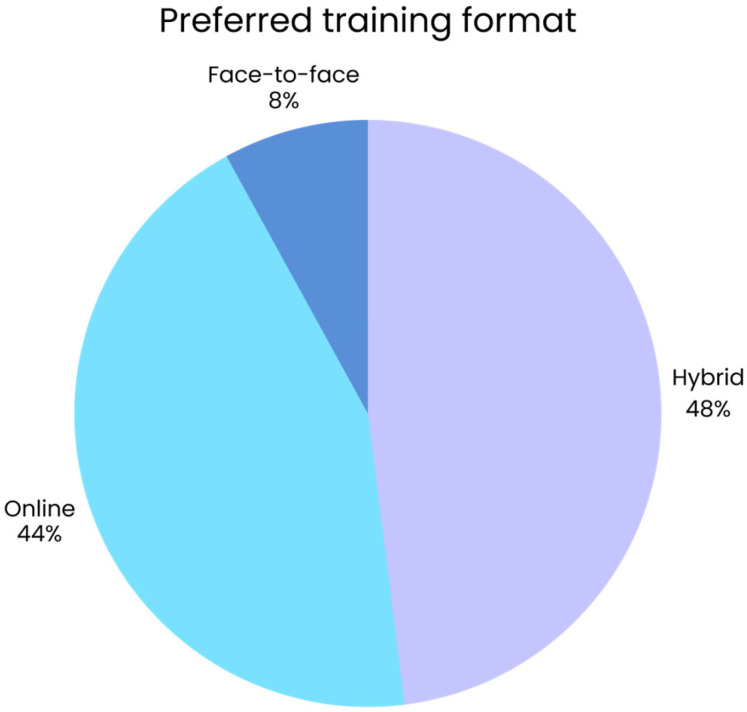
Preferred training format among family doctors (*n* = 50).

**Figure 4:**
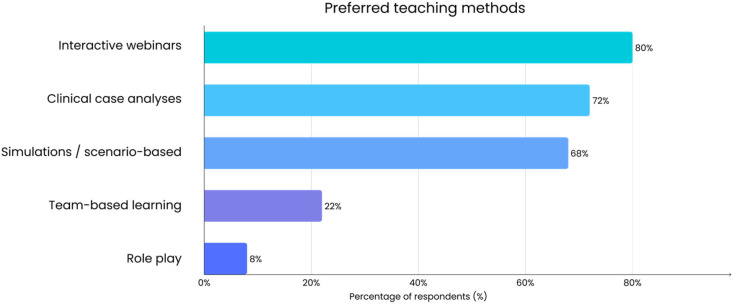
Preferred teaching methods for cancer prevention training among family doctors (*n* = 50). Multiple responses were allowed for this survey question, so percentages do not sum to 100%.

Participants also indicated how they would prefer future training to be delivered. As shown in Figure 3, flexible learning formats were clearly favored, with blended and online approaches preferred over traditional face-to-face sessions. In addition to format, respondents expressed clear preferences regarding teaching methods. As shown in Figure 4, interactive, case-based, and simulation-based approaches were strongly favored, whereas role-play, team-based projects, and community outreach received little interest.

#### 3.2.4. Collaboration with Cancer Centers

**Figure 5:**
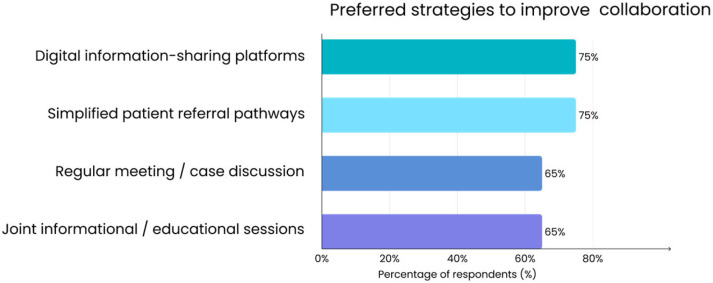
Preferred strategies to improve collaboration between family doctors and cancer centers (*n* = 50). Multiple responses were allowed for this survey question, so percentages do not sum to 100%.

Surveyed physicians also indicated preferred strategies to improve collaboration with cancer centers. As shown in Figure 5, the most frequently mentioned solutions were digital information-sharing platforms and simplified referral pathways. In addition, many respondents reported regular meetings or case discussions as useful, and a similar proportion selected joint informational or educational sessions organized by cancer centers.

### 3.3. Subgroup Analyses

#### 3.3.1. Urban vs. Rural Practices

Overall, urban and rural family doctors responded similarly on most survey items, with one notable exception related to training format preferences. A chi-square test showed a significant association between practice location and preferred training delivery mode (*p* < 0.05). Rural physicians were much more likely to favor fully online training compared to their urban counterparts, whereas urban physicians more often preferred a blended format. Specifically, half of rural doctors (50%) chose an online-only format as their top choice (versus 42% of urban doctors), and most urban doctors (56%) opted for mixed/hybrid training (versus 29% of rural doctors). Exclusively face-to-face training was selected by a small minority, more frequently among rural physicians (21%, *n* = 3) than urban physicians (3%, *n* = 1).

Aside from training logistics, rural and urban respondents did not differ significantly in their interest in training content or in most prevention practices. Both groups were almost universally interested in participating in further training (roughly 95% in each group said “yes” to training, with no significant difference). They also had nearly identical topic preferences—high interest across the board—and reported similar behaviors regarding lifestyle counseling frequency and use of preventive tools. For example, rural doctors were just as likely as urban doctors to recommend advanced tests like tumor markers or genetic screening when available (no significant urban-rural gap in the use of these analyses was found). No significant urban-rural differences were observed in prevention-related practices or training content interest, suggesting that these attitudes and needs are consistent across practice locations. The primary adjustment to consider is in how training is delivered to accommodate those in rural practice.

#### 3.3.2. Differences by Years of Experience

We also compared responses among physicians with <5 years, 5–10 years, and >10 years of practice to see if newer versus more veteran doctors differed in perspectives. In general, there were no major statistically significant differences across these experience groups on key outcomes (one-way ANOVA and chi-square tests, all *p* > 0.1). Family doctors of all experience levels showed a consistently high commitment to prevention and an eagerness for training (high proportions across all groups reported interest in lifestyle counseling and further education). Descriptive differences were observed between groups, but these did not reach statistical significance.

Familiarity with the emerging sciences was generally moderate across all experience groups. Physicians with less than 5 years of practice reported the highest average score (M = 2.5 on a 1–4 scale, where 1 = not at all and 4 = very familiar). Those with 5–10 years of practice had the lowest average (M = 2.0), while physicians with more than 10 years reported a similar mean (M = 2.03). None of the early-career doctors indicated being completely unfamiliar with omic science, whereas among the most experienced group almost a quarter selected this option. Awareness of the European Code Against Cancer also varied by experience. All physicians with less than 5 years of practice reported at least partial awareness, while among those with more than 10 years, 38% indicated no awareness of the Code. Early-career physicians reported higher familiarity with omic sciences and greater awareness of recent European recommendations, but this difference was not supported by statistical significance (*p* > 0.1).

When asked about the concept of personalized prevention, physicians across all experience groups reported moderate levels of familiarity. Physicians with more than 10 years of practice reported the highest average score (M = 2.6 on a 1–4 scale), while those with less than 5 years and those with 5–10 years reported slightly lower means (M = 2.3 and M = 2.2, respectively). In the group with over 10 years of experience, 15% indicated knowing the concept “very well,” compared to none in the <5-year group. Although more experienced physicians tended to rate their familiarity higher, statistical testing showed no significant differences between groups (*p* > 0.1).

## 4. Discussion

### 4.1. Barriers to Delivering Preventive Care

A primary barrier hindering effective cancer prevention in primary care is the significant time constraints faced by family physicians. Family doctors often prioritize acute care pressures over preventive practices due to the urgent nature of patients’ complaints during consultations. Findings indicate that a significant proportion of surveyed physicians cited insufficient consultation time as a core obstacle, corroborating previous studies that have found that physicians often feel they lack adequate time to thoroughly engage with patients on cancer preventive measures [15,16]. Family physicians expressed that while they recognize the importance of preventive care, the demands of a busy schedule restrict their ability to discuss these topics in any meaningful detail. 

Beyond time constraints, systemic hurdles further exacerbate the challenges associated with implementing preventive strategies. Many physicians reported issues regarding the healthcare system’s reimbursement policies, emphasizing that a lack of financial incentives for preventive services dissuades them from prioritizing these activities. The assertion that healthcare systems do not adequately compensate for preventive care was echoed by physicians across various studies, including those identifying structural inefficiencies in billing and reimbursement for prevention efforts [16]. These systemic barriers are not unique to our setting; similar challenges regarding reimbursement mechanisms, workload, and diagnostic access have been reported in broader European policy analyses [17]. Additionally, with the burdensome administrative workloads corresponding to Health Record usage consuming significant portions of clinical time, physicians often find themselves torn between fulfilling documentation requirements and engaging in valuable patient interactions [18].

Moreover, patient-related barriers pose serious challenges for family doctors. Many patients exhibit low health literacy and are often influenced by misinformation regarding cancer prevention, creating obstacles to effective communication. Approximately 62% of surveyed physicians indicated that a lack of accessible information for patients limits their ability to facilitate preventive measures, a finding consistent with previous studies [19,20]. Consequently, family physicians are frequently required to counteract patients’ fears and misconceptions, detracting from time that could otherwise be spent on preventive counseling.

### 4.2. Solutions to Overcome Barriers

To mitigate these barriers, a multifaceted approach involving organizational, provider-focused, and patient-centered strategies is required. Implementing organizational solutions such as reminder systems can enhance preventive care uptake by prompting patients to attend necessary screenings and consultations. For example, studies suggest that utilizing clinical decision support (CDS) tools in primary care enhances the identification and documentation of appropriate preventive measures during patient interactions [21]. The integration of technology can streamline processes, reduce administrative burdens, and allow for greater focus on patient care during appointments.

Provider-focused solutions also need to be prioritized, especially concerning ongoing education and training in cancer prevention. The palpable enthusiasm for training on cancer prevention evident among physicians suggests a significant opportunity to provide structured educational resources that cater to their needs. Interactive and case-based continuing medical education (CME) is particularly beneficial, as it has shown to increase the competency of family physicians in preventive counseling [22]. Incorporating new scientific knowledge into practical training modules would not only empower physicians but also streamline the integration of concepts like genomic risk assessment into routine care delivery.

### 4.3. Training, Education and Health Literacy in Cancer Prevention

Gaps in knowledge and shifting paradigms in medicine necessitate a continuous reassessment of clinicians’ training and continuing education. Recent literature emphasizes that primary care involves increasingly complex preventive care processes and, thus, requires updated educational curriculums that include emerging trends such as personalized medicine [16,23]. Physicians expressed a strong interest in further training not only on cancer risk factors but also on effective patient communication about these complex issues. Creating tailored education programs focused on the nuances of personalized cancer risk would ultimately boost the competence of family doctors and enhance patient understanding and engagement. A practical and cost-effective solution would be to integrate such content into existing continuing medical education programs or to pilot blended training modules within national CME frameworks.

Moreover, adopting a holistic view of patient care that champions preventive strategies will likely lead to significant improvements in health outcomes. Numerous studies emphasize the overarching benefits of empowering patients through education, motivating them to seek screenings and preventative measures actively [16,23]. Addressing misinformation, improving health literacy, and fostering meaningful dialogs between patients and providers can collectively transform preventive care practice, allowing family physicians to fulfill their ideal roles as proactive guardians of public health.

### 4.4. Integration into Expertise Networks for Prevention

Another beneficial strategy includes fostering stronger collaborations between family medicine and specialized oncology services. Establishing formal networks can enhance communication between primary care providers and oncologists, allowing for the better integration of patient care. Physicians have voiced the need for the improved continuity of information and feedback about referred patients, with many expressing frustration at receiving little follow-up from specialists post-referral, a concern similarly highlighted in other studies [18,24]. Creating platforms wherein primary care and specialized cancer teams can readily exchange insights would facilitate coordinated patient management and bolster detection efforts for high-risk patients.

The advantages of multidisciplinary collaboration extend beyond mere communication. Studies suggest that such partnerships can significantly improve screening rates and early detection of cancer cases [25]. For instance, integrated models have demonstrated the efficacy of collective expertise in identifying individuals at elevated risk for cancers, ultimately ensuring that preventive services are efficiently delivered [19]. This integrated approach could enhance physicians’ understanding of contemporary screening guidelines, ultimately translating to better patient outcomes as family physicians remain informed and engaged throughout their patients’ journeys. Future research should explore the implementation of these collaborative solutions and evaluate their impact on preventive outcomes.

### 4.5. Limitations

This study presents several limitations. First, the qualitative and quantitative data were primarily collected from family physicians practicing in Cluj County, Romania, particularly in urban settings, which may limit the generalizability of findings to other regions or rural practices. Second, the relatively low response rate to recruitment emails could suggest potential self-selection bias, with participants possibly representing family doctors who are inherently more engaged or interested in preventive care. Moreover, subgroup analyses should be interpreted with caution given the limited sample size and reduced statistical power, and their findings regarded as exploratory. The cross-sectional nature of the quantitative survey restricts the ability to infer causality or assess changes in perceptions and practices over time. Future studies should consider broader geographic sampling, longitudinal designs, and strategies to enhance participant response rates.

## 5. Conclusions

This mixed-methods study highlighted important insights regarding the role, challenges, and educational needs of family physicians in cancer prevention within the context of primary healthcare. Family physicians recognize their critical role in preventive medicine; however, several interconnected barriers significantly limit their ability to implement optimal preventive practices. These barriers include limited consultation time, patient-related issues such as misinformation and health literacy deficits, financial constraints, and systemic limitations including inadequate reimbursement and cumbersome administrative workloads. Our findings align with international evidence, underscoring that these challenges are widespread rather than isolated occurrences.

To address these barriers, multifaceted interventions are required. Promising solutions identified in our study and supported by existing literature include organizational enhancements such as reminder systems and clinical decision-support tools; structured, interactive, and practical continuing medical education programs tailored specifically to cancer prevention and patient counseling; and multidisciplinary collaborative models integrating primary care providers with cancer specialists through formal expertise networks.

The integration of personalized medicine, omics sciences, and advanced screening technologies into primary care represents another opportunity, though current adoption is limited by knowledge gaps and resource availability. Therefore, targeted education and training are essential to empower family physicians to leverage these advances effectively.

## Data Availability

The datasets generated and analyzed during the current study are available from the corresponding author on reasonable request. The data are not publicly available due to privacy and ethical restrictions.

## Appendix A

The following guide presents the semi-structured interview questions used to explore family physicians’ perspectives on their role in cancer prevention, patient counseling, and collaboration with other specialists.

Context:

1. What are the main roles of the family physician in the healthcare system?
2. What current activities does the family physician carry out in the practice?
3. How do you assess the current reimbursement of medical services?
4. How can the family physician be involved in the prevention network for oncological diseases?
5. How can the family physician be supported by other specialists (oncologists, internists, geneticists, public health physicians, etc.) in developing personalized cancer prevention plans?

Counseling/Education:

6. How do you see the role of the family physician in patient education?
7. What difficulties do you encounter in communicating with patients?
8. What are the most frequent lifestyle recommendations you provide?
9. What medications or vaccines do you recommend for the prevention of certain types of cancer?
10. What additional resources and information do you provide to patients regarding healthy lifestyle and cancer prevention?

Oncological Screening:

11. How is the risk of developing oncological diseases assessed?
12. What are the most common cancers for which you perform/recommend screening?
13. What tests or analyses necessary for the detection of oncological diseases do you recommend?
14. How do you assess genetic factors in cancer prevention (especially within the family of a cancer patient)?
15. Is there a different approach to cancer prevention and detection in asymptomatic patients compared to those with chronic conditions?

## References

[1] Sung H., Ferlay J., Siegel R.L., Laversanne M., Soerjomataram I., Jemal A., Bray F. (2021). Global Cancer Statistics 2020: GLOBOCAN Estimates of Incidence and Mortality Worldwide for 36 Cancers in 185 Countries. CA A Cancer J. Clin..

[2] Wild C.P., Espina C., Bauld L., Bonanni B., Brenner H., Brown K., Dillner J., Forman D., Kampman E., Nilbert M. (2019). Cancer Prevention Europe. Mol. Oncol..

[3] Yu S., Cai X., Wang X., Lin X., Cai S. (2024). Disease burden of breast cancer and risk factors in Europe 44 countries, 1990-2019: Findings of the global burden of disease study 2019. Front. Endocrinol..

[4] Casali P.G., Antoine-Poirel H., Berrocoso S., Blay J.Y., Dubois T., Ferrari A., Fullaondo A., Hovig E., Jagodzińska-Mucha P., Ługowska I. (2025). Health networking on cancer in the European Union: A ‘green paper’ by the EU Joint Action on Networks of Expertise (JANE). ESMO Open.

[5] Klepp K.I. (2023). JAPreventNCD. Eur. J. Public Health.

[6] Pastorino R., Pezzullo A.M., Osti T., Adany R., Borry P., Barnhoorn F., Fadil E., Kroese M., Metspalu A., Perez-Gomez B. (2024). The PROPHET project paves the way for personalized prevention in the future healthcare. Eur. J. Cancer Prev..

[7] Liang J., Lin Y., Liang H., Qi J., Ni J., Lin H., He J. (2024). Intercontinental and Regional Disparities in Cancer Burden: A Comprehensive Analysis of Trends and Projections Using 1990–2021 GBD Data with Forecasting to 2035. medRxiv.

[8] Ebling B., Juretić A., Pribić S., Ebling Z. (2024). National colorectal cancer early detection program and colorectal cancer early detection model integrated in the practice of family medicine: Our comparison. Libr. Oncol. Croat. J. Oncol..

[9] Elagi A.A., Jaber B., Wassly A.A., Ahmed R.S., Bosily F.A. (2019). Public’s perception and satisfaction on the role and services provided by family physicians in Saudi Arabia: A cross-sectional study. J. Fam. Med. Prim. Care.

[10] Ciuba A., Wnuk K., Nitsch-Osuch A., Kulpa M. (2022). Health Care Accessibility and Breast Cancer Mortality in Europe. Int. J. Environ. Res. Public Health.

[11] Alzaben A.S., Aljahdali A.A., Alasousi L.F., Alzaben G., Kennedy L., Alhashem A. (2023). Nutritional Knowledge, Attitudes, and Practices among Family Physician Practitioners in Gulf Countries (Bahrain, Kuwait, Saudi Arabia, and UAE). Healthcare.

[12] Harry M.L., Truitt A.R., Saman D.M., Henzler-Buckingham H.A., Allen C.I., Walton K.M., Ekstrom H.L., O’Connor P.J., Sperl-Hillen J.M., Bianco J.A. (2019). Barriers and facilitators to implementing cancer prevention clinical decision support in primary care: A qualitative study. BMC Health Serv. Res..

[13] Milićević M.Š., Djurin A., Terzić-Šupić Z., Todorović J., Nikolić D., Soldatović I. (2022). Knowledge and barriers to early detection of breast cancer among female primary care patients in Serbia. Cent. Eur. J. Public Health.

[14] DeJonckheere M., Vaughn L.M. (2019). Semistructured interviewing in primary care research: A balance of relationship and rigour. Fam. Med. Community Health.

[15] Saman D.M., Chrenka E.A., Harry M.L., Allen C.I., Freitag L.A., Asche S.E., Truitt A.R., Ekstrom H.L., O’Connor P.J., Sperl-Hillen J.M. (2021). The impact of personalized clinical decision support on primary care patients’ views of cancer prevention and screening: A cross-sectional survey. BMC Health Serv. Res..

[16] Samimi G., Douglas J., Heckman-Stoddard B.M., Ford L.G., Szabo E., Minasian L.M. (2022). Report from an NCI Roundtable: Cancer Prevention in Primary Care. Cancer Prev. Res..

[17] Kostadinov K., Iskrov G., Musurlieva N., Stefanov R. (2025). An evaluation of rare cancer policies in Europe: A survey among healthcare providers. Cancers.

[18] Milley K., Druce P., McNamara M., Bergin R.J., Chan R.J., Cust A.E., Davis N., Fishman G., Jefford M., Rankin N. (2024). Cancer in general practice research priorities in Australia. Aust. J. Gen. Pract..

[19] Saman D.M., Walton K.M., Harry M.L., Asche S.E., Truitt A.R., Henzler-Buckingham H.A., Allen C.I., Ekstrom H.L., O’Connor P.J., Sperl-Hillen J.M. (2019). Understanding primary care providers’ perceptions of cancer prevention and screening in a predominantly rural healthcare system in the upper Midwest. BMC Health Serv. Res..

[20] Winarto H., Mitzy B.S., Widodo A.B., Kurniawan A., Phallaphi Y.R. (2019). Cervical Cancer Related Knowledge, Attitude and Behaviour Among Women in Makasar District Primary Health Care Centre in 2018. Open Public Health J..

[21] Sperl-Hillen J.M., Rossom R.C., Kharbanda E.O., Gold R., Geissal E.D., Elliott T.E., Desai J.R., Rindal D.B., Saman D.M., Waring S.C. (2019). Priorities Wizard: Multisite Web-Based Primary Care Clinical Decision Support Improved Chronic Care Outcomes with High Use Rates and High Clinician Satisfaction Rates. EGEMs (Gener. Evid. Methods Improv. Patient Outcomes).

[22] Kok M.Y., Chavez J.C., Quesada P.R., Adegoke O.T., Chang S. (2022). Pathways and Barriers to Careers in Academic Clinical Cancer Prevention: A Qualitative Study. J. Cancer Educ..

[23] Hashemi N., Bahrami M., Tabesh E. (2023). Development, implementation, and evaluation of a program to expand the nurse’s roles in colorectal cancer prevention: A mixed-methods protocol study. J. Educ. Health Promot..

[24] Wood M.E., Rehman H., Bedrosian I. (2020). Importance of family history and indications for genetic testing. Breast J..

[25] Schlueter D., DeGroff A., Soloe C., Arena L., Melillo S., Tangka F., Hoover S., Subramanian S. (2023). Factors That Support Sustainability of Health Systems Change to Increase Colorectal Cancer Screening in Primary Care Clinics: A Longitudinal Qualitative Study. Health Promot. Pract..

